# Just One Move: co-design and usability testing of a digital physical activity behavior change platform for people with rheumatoid arthritis

**DOI:** 10.3389/fpubh.2026.1972848

**Published:** 2026-10-29

**Authors:** Manuel Ester, Daniel Gillespie, Kiran Dhiman, Krista White, Melissa Sipley, Daniyal Sait, Aidan Lee, Racheal Githumbi, Nikki Bhatti, Eileen Davidson, Linda L. Roy, Carole Silvera, Kamala Adhikari, Claire E. H. Barber

**Affiliations:** 1 McCaig Institute for Bone and Joint Health, Cumming School of Medicine, University of Calgary, Calgary, AB, Canada; 2 O’Brien Institute for Public Health, Cumming School of Medicine, University of Calgary, Calgary, AB, Canada; 3 Arthritis Research Canada, Vancouver, BC, Canada; 4 Department of Physical Therapy, Faculty of Rehabilitation Medicine, University of Alberta, Edmonton, AB, Canada; 5 Division of Rheumatology, Cumming School of Medicine, Calgary, AB, Canada; 6 Department of Community Health Sciences, Cumming School of Medicine, University of Calgary, Calgary, AB, Canada; 7 Primary Care Alberta, Calgary, AB, Canada; 8 School of Public Health, University of Alberta, Edmonton, AB, Canada; 9 Department of Medicine, Cumming School of Medicine, University of Calgary, Calgary, AB, Canada

**Keywords:** co-design, eHealth, exercise, health behavior change, patient-oriented research, physical activity, rheumatoid arthritis, website

## Abstract

**Introduction:**

Rheumatoid Arthritis (RA) affects 18 million people worldwide, leading to debilitating symptoms and increased cardiovascular disease risk. Regular physical activity can address these challenges, yet people with RA face barriers to physical activity and lack tailored support. Digital interventions offer a resource-efficient strategy for increasing physical activity. To improve their effectiveness and implementation, stronger end-user engagement and behavioral science integration is needed. Alongside physical activity experts, rheumatology providers, and people with RA, we completed preparatory research to inform the co-design of an RA-specific digital physical activity intervention: JustOneMove.ca. The present study presents learnings from the co-design process, outlines the resultant intervention, and summarizes usability survey findings.

**Methods:**

The study leveraged patient-oriented research, user-centered design, and behavioral science frameworks (behavior change wheel, theoretical domains framework). After mapping physical activity barriers to these frameworks, Just One Move was co-designed via a 4-step user-centered process (Phase 1). We collaborated with people with RA and web design experts to review, design, refine, and develop the intervention using design sprint methodology. Data on resources required and partner involvement during Phase 1 were summarized descriptively. A mixed methods survey (Phase 2) gathered perspectives on usability and usefulness. Data were analyzed using descriptive statistics and qualitative content analysis.

**Results:**

Co-design was completed in 7 months by a team of 20 people with RA, 5 researchers (rheumatology, physiotherapy, behavioral science), and 2 experts in healthcare design and web development. Co-design blended research evidence and user preferences through design thinking. Challenges included balancing creativity with feasibility and defining clear expectations. The Just One Move intervention includes: a library of RA-friendly movements, a guided action planner, a collection of inspiring stories from people with RA, and an expert toolkit with tips for moving well with RA. Across 37 users (median age: 62.0), average usability was 4.0/5 and usefulness was 3.7/5. Just One Move was easy to use and inspired confidence to move more. However, users requested clearer instructions and more customizable content.

**Conclusion:**

Evidence-informed co-design can produce high-quality digital physical activity interventions for people with RA. Future work will examine the effectiveness of JustOneMove.ca.

## 1 Introduction

Rheumatoid arthritis (RA) is a common type of inflammatory arthritis, affecting over 18 million people worldwide. Despite significant pharmacological treatment advances to control disease activity, many people with RA experience frequent inflammatory flares, debilitating pain and fatigue, reduced function and overall well-being, and increased risk of secondary chronic conditions (1–3). Regular physical activity can address common symptoms, attenuate functional decline, reduce the risk of cardiovascular disease, and improve quality of life for people with RA (4, 5). This could save the healthcare system millions (6).

International guidelines recommend the integration of physical activity into standard rheumatology care, yet people with RA remain less active than the general population and rarely receive physical activity advice or referrals from their care team (7–11). They face significant disease-related (fatigue, joint pain), psychosocial (fear and uncertainty about safe activities), and environmental (lack of RA-specific programs, insufficient professional support) barriers to physical activity (12, 13). Furthermore, the implementation of physical activity interventions in rheumatology lags far behind other chronic conditions (cardiovascular disease, diabetes, osteoarthritis), where standard care includes evidence-based physical activity programs (14–16).

There is an urgent need to co-design and implement effective, RA-specific physical activity interventions in rheumatology care. Digital behavior change interventions represent a potential resource-efficient strategy for increasing physical activity among people with RA (17). However, their effectiveness and implementation remains sub-optimal. This may be in part due to the limited use of behavioral science theory and end-user engagement strategies during intervention development (18–20). The United Kingdom Medical Research Council (MRC) and National Institute for Health and Care Research (NIHR) guideline to developing complex interventions emphasizes the importance of using behavioral science theories and intervention co-design to enhance real-world uptake and effectiveness (21). Engaging end-users through a co-design approach improves contextual tailoring of interventions to real-world needs and preferences, seeking to overcome challenges related to intervention engagement and real-world implementation (22–24). Traditional top-down development approaches driven by a small team of experts may miss these important contextual nuances. Involving people with RA and their healthcare providers in the process also builds a sense of ownership and trust towards outputs (25).

We co-designed Just One Move, an RA-specific digital physical activity intervention, using an integrated knowledge mobilization (iKMb) approach alongside the United Kingdom MRC/NIHR guideline (26). Co-design was defined as active collaborative with interest holders to design a solution to physical inactivity among people with RA (23). The iKMb framework ensured that relevant patient and provider needs, preferences, and contextual nuances were considered during intervention co-design, and to inform the subsequent usability evaluation (26). Specifically, people with RA and other relevant partners were engaged throughout all phases of the work, with 3 additional core elements from the MRC/NIHR guideline selected as critical for the co-design process (consider context, develop refine and retest programme theory, and identify uncertainties).

The target population for Just One Move are insufficiently active people with well-controlled RA who are looking for a self-led digital support program. To lead the co-design of Just One Move, we first built a core team of researchers, rheumatology care providers, and people with RA. This core team completed an umbrella review of physical activity behavior change in inflammatory arthritis, environmental scan and critical appraisal of existing online RA-specific physical activity resources, and priority setting to identify the most important physical activity barriers and facilitators for people with RA (i.e., Phase 0: Preparation in Figure 1) (9, 12, 27, 28). Findings from the preparatory phase provided a strong evidence-based, people-centered foundation that directly informed Just One Move co-design.

**Figure 1:**
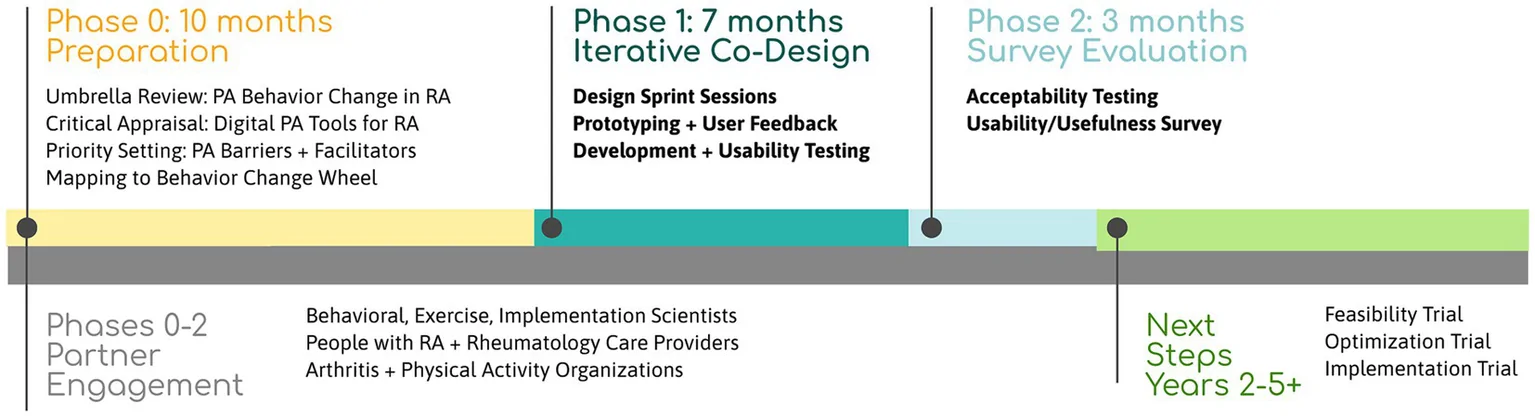
Overview of Just One Move co-design phases, including preparatory work and next steps. Phases 1 and 2 are described in this paper. PA, physical activity.

The aims of the present manuscript are to describe the iterative co-design process, present the resultant Just One Move intervention, and summarize the mixed methods findings from our usability testing survey. This work represents a valuable example for co-developing evidence-based, patient-centered physical activity interventions for RA and other chronic conditions.

## 2 Methods

### 2.1 Guiding principles, theories, models, and frameworks

The study was informed by guiding principles from patient-oriented research, user-centered design, as well as behavioral science (29–31). Intervention co-design was informed by the behavior change wheel (BCW), consisting of the Capability, Opportunity, Motivation model of behavior change, theoretical domains framework, and evidence-based intervention functions, and the behavior change techniques taxonomy (BCT) (31, 32). Priorities identified by people with RA during the preparation phase were mapped to the BCW and respective Theoretical Domains Framework domains, before matching them to evidence-based intervention strategies and BCTs using the BCW and theory and techniques tool [12, 33. The behavioral mapping of patient priorities was completed prior to starting intervention co-design and is described in detail elsewhere (12).

The BCW and Theoretical Domains Framework was selected for its strong roots in physical activity behavior change science, and evidence-based linkage between behavioral determinants and intervention strategies to address them. The results of our behavioral mapping served as the foundation for co-design, keeping the intervention grounded in core patient-derived behavioral determinants as well as evidence-based intervention strategies and BCTs (12).

An iterative, user-centered co-design process was carried out alongside people with RA and rheumatology providers, transforming intervention strategies and selected BCTs into user-friendly intervention components. Co-design was aligned with 8 common principles the past 10 years of co-design in public health research: inclusive, participative, equal, supportive, applicable, iterative, cooperative, and transparent (24).

Briefly, the approach was inclusive, participative, and equal by purposively sampling a diverse sample of people with RA and care providers as equal partners, providing opportunities for participation using consensus techniques and co-design workshops across the research. A supportive environment was fostered by adapting scheduling to partner needs, providing synchronous and asynchronous ways to provide feedback, and offering fair compensation. Strong cooperation was built using introductory meetings and open communication to build rapport, with iterative intervention refinement driven by partner feedback. Feedback and design decision summaries were shared with partners for transparency, followed by prototyping and usability testing later in the process to ensure solutions were applicable to the people and contexts they intended to serve. Further details on the co-design process are provided in section 2.5.

### 2.2 Partner engagement

Patient partners with lived expertise of RA were involved throughout the preparatory, co-design, and usability testing phases (see Figure 1) (9, 12, 27, 28). They co-led the development of our digital health intervention in phase 1, shaping the design process and contributing to decision making. Our patient partners ensured that the final digital intervention addressed key needs and preferences of people with RA with regards to usability and usefulness. Individual meetings were held with each patient partner prior to phase 1 to develop a shared understanding of roles, preferences, needs, and expectations. Patient partners were remunerated for their time according to CIHR strategy for patient-oriented research guidelines (33). At the project mid-point, patient partners reported on the quality of their involvement using the PEIRS-22 questionnaire, a validated tool for assessing patient engagement in research (34). These data were reviewed internally to adapt partner engagement approaches for the latter half of the project. Additional information is provided throughout the manuscript and summarized in Supplementary Table 1 (35).

### 2.3 Study design

This 2-phase study included the iterative patient-driven intervention co-design process (phase 1), followed by a cross-sectional mixed methods usability testing survey (phase 2). Ethical approval was obtained through the University of Calgary (REB24-0858).

### 2.4 Participants and recruitment

Two independent groups of Canadian adults with RA were recruited to participate in either phase 1 or phase 2. This included anyone with a confirmed diagnosis of RA who was (1) at least 18 years of age, (2) currently living in Canada, and (3) able to participate in English. Eligible individuals were recruited through email invites sent to past research participants who agreed to be contacted about future research and members across various partner organizations (Arthritis Research Canada, Canadian Arthritis Patient Alliance). To avoid response bias between phases, participants in phase 1 were not eligible to respond to the usability testing survey in phase 2. Participants in phase 1 provided verbal consent to participate as patient research partners after reviewing the terms of reference with a member of the research team. Participants in phase 2 provided written informed consent prior to completing the usability testing survey.

### 2.5 Phase 1: co-design of the digital physical activity behavior change intervention

A visual overview of the co-design process is shown in Figure 2. The user-centered process was carried out across 4 steps: (1) Review, (2) Design, (3) Refine, (4) Develop. Steps 2–4 were supported by a 2-person design team, which included an expert in healthcare design thinking from the Alberta Health Services Design Lab, with over 10 years of experience in designing healthcare innovations provincially using design thinking, systems thinking, service design, codesign, and strategic foresight, and an independent brand strategy and web development expert.

**Figure 2:**
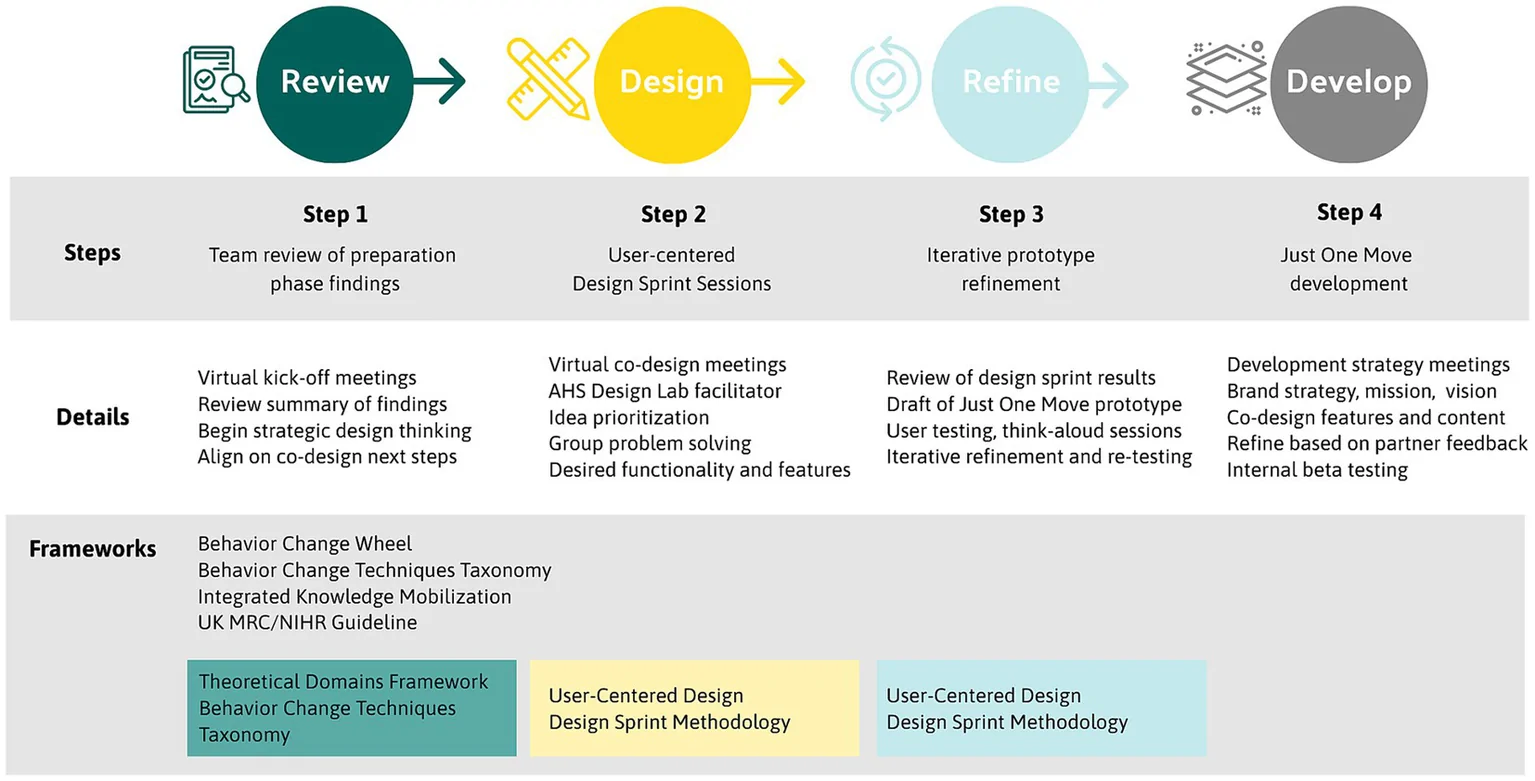
The 4 steps of the Just One Move co-design process.

#### 2.5.1 Step 1 (review): team review of preparation phase findings

During this step, 14 patient partners and 4 research team members met across 2 virtual co-development kick-off meetings. Each 1-h meeting was scheduled according to patient partner availability. The meeting agenda included 3 aims to (1) present a brief practical summary of findings to date, (2) initiate strategic thinking to address physical activity determinants using identified intervention strategies and BCTs, and (3) develop a shared understanding of next steps in the co-design process.

#### 2.5.2 Step 2 (design): user-centered design sprint sessions

In collaboration with the Alberta Health Services Design Lab, a facilitation agenda was developed for creative brainstorming sessions using Google’s design sprint methodology (Supplementary File 1) (36). The sessions focused on the middle stages of the design sprint process, sketch and decide, generating a broad range of creative ideas before narrowing them down as a team towards a clear concept for prototyping. A summary of preparatory work and meeting notes from Phase 0 were shared with the design team to tailor session structure. Each session was facilitated by the Design Lab, guiding participants through a series of activities to refine their creative ideas for the digital physical activity intervention. The session started with prioritizing ideas from preparatory phase findings (Card Sorting), prompted blue sky thinking about solving the problem (How Might We), asked participants to speak about related challenges (Analogies), followed by an open brainstorming on functionality and features (The Big Storm) and discussion about which ideas to focus on (Prioritization). See Supplementary File 1 for more details. Meeting notes were summarized by the research team with the help of Zoom AI note-taking tools. They were shared with all team members, including the design team and patient partners, gathering additional feedback to inform next steps.

#### 2.5.3 Step 3 (refine): iterative prototype refinement

After reviewing the key ideas from the design sprint sessions, research and design teams met to draft the first prototype of our digital physical activity intervention, Just One Move. After initial refinement, a working prototype was created using Microsoft PowerPoint, allowing users to interact with the product in real time like a real website (Supplementary Figure 1). Next, patient partners were invited to participate in 1–1 usability testing sessions, where they were asked to complete a series of “think-aloud” tasks using the prototype and describe their thought processes as well as initial impressions. A member of the research team recorded detailed notes during each session, leading to iterative refinement. An *a priori* end point of 10 usability testing sessions was selected to capture a diverse range of user experiences, unless major concerns emerged during the final few sessions. Additional information for this step is available in Supplementary File 2.

#### 2.5.4 Step 4 (develop): just one move development

Upon completion of user testing, the working prototype and a feedback summary from steps 1–3 was shared with our web developer to begin Just One Move development. The web developer then led a series of 6 strategy meetings with the research team to clarify the mission and vision for our digital intervention, agree on a name and brand strategy, choose a look and feel, and refine the technical details as well as intervention content. Content requirements for all Just One Move components were detailed in a master development plan. The research team collaborated to develop content over a period of 3 weeks. Patient partners, exercise professionals, behavioral scientists, and rheumatology clinicians provided advisory oversight during this step. Partners were invited via email to provide critical feedback on strategy documents and website page previews between strategy meetings. This feedback was integrated into meeting notes or discussed at subsequent meetings. After all content was added to the Just One Move platform, the research team and key partners (patient partners and rheumatologists from earlier co-design phases) conducted final internal tests for functionality and content accuracy. Errors and final tweaks for content and functionality were reported directly to the developer using a reporting spreadsheet and resolved prior to launch.

### 2.6 Phase 2: preliminary usability and usefulness testing

A feedback survey was launched to collect preliminary data on the usability and usefulness of Just One Move. The survey instructed participants to spend at least 15 min familiarizing themselves with all elements of the Just One Move, before returning to answer a series of questions regarding their initial impressions. The brief survey contained standard demographics questions including age, years living with RA, gender, race, and education level, self-reported physical activity (GLTEQ: Godin Leisure Time Exercise Questionnaire), and physical activity self-efficacy (PA SES: Physical Activity Self-Efficacy Scale) (37, 38). The GLTEQ prompts participants to report the frequency of mild (minimal effort), moderate (not exhausting), and strenuous (heart beats rapidly) physical activity in bouts of 15 min or more during a typical week (34). A validated tool was used to assess usability and usefulness of the intervention (MARS: Mobile App Rating Scale, language adapted for website format), alongside three optional open-ended questions (“What did you like?” “What did you dislike?” “What could be improved?”) to provide feedback on overall impressions (39). The MARS features 29 items across 6 domains: engagement, functionality, aesthetics, information, subjective quality, and perceived impact, each evaluated using a 5-point Likert scale (1 = inadequate to 5 = excellent) (39).

### 2.7 Data collection and analysis

During Phase 1, data were recorded on the type and number of participants involved in each step of the co-design process. These data were summarized descriptively to understand the level of involvement of various interest holders during the process, as well as the time invested in co-design. Quantitative data from Phase 2, including demographics, self-reported physical activity levels and self-efficacy, and MARS scores were analyzed in R to calculate descriptive summary statistics. For categorical data, frequencies and percentages were calculated. For continuous data, means and standard deviations were calculated for normally distributed variables, with medians and interquartile ranges used for non-normal data. The GLTEQ, PA SES, and MARS results were processed and analyzed according to guidelines provided by the respective developers (37, 39). For the GLTEQ, the Leisure Score Index was calculated using the following formula: light physical activity x 3 + moderate physical activity x 5 + strenuous physical activity x 9. Scores were classified as insufficiently active (below 14), moderately active (14–23), or active (above 23) using GLTEQ scoring guidelines (37). As per the MARS analysis guidelines, an overall quality score was calculated by averaging scores across engagement, functionality, aesthetics, and information (38). Quality (i.e., average of items 1–19) and subjective quality (i.e., average of items 20–23) scores are independent. All quantitative analyses were completed using R version 4.5.2. Open-ended survey feedback was imported into NVivo and analyzed using qualitative content analysis by the first author (ME) (40). Qualitative feedback was reviewed across 2 team meetings, with co-authors providing feedback to refine the qualitative findings.

## 3 Results

### 3.1 Phase 1: iterative co-design

Co-design process: timeline and resources.

The co-design of our digital physical activity intervention, JustOneMove.ca, was completed during a 7-month period from November 2024 to July 2025. The co-design team included 20 people with lived expertise of RA, each of whom participated in 1–4 steps of the process, 5 research team members, including a rheumatologist, physiotherapist, and physical activity behavior change expert, and 2 external collaborators specializing in healthcare design and web development. This period included 30 meetings with an average duration of 60 min, amounting to 30 total hours (Supplementary Table 2).

### 3.2 Co-design results: overview of just one move

An overview of Just One Move features, including name, purpose, and associated BCTs is provided in Figure 3. Screenshots for the landing page and additional features are shown in Figures 4 and 5 below. All decisions were informed by patient partner feedback during the co-design process and supported by evidence from our phase 0 preparatory work as well as physical activity behavior change literature (9, 12, 27–32, 41).

**Figure 3:**
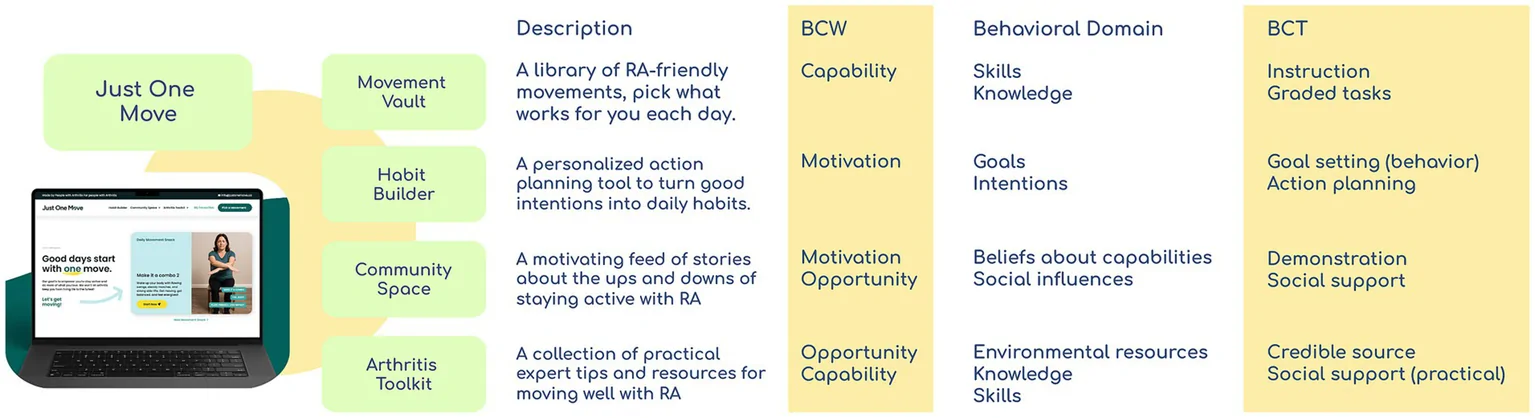
Overview of Just One Move features. BCW, Behavior Change Wheel. BCT, Behavior Change Techniques. This figure contains AI-generated individuals. No real photos were used.

**Figure 4:**
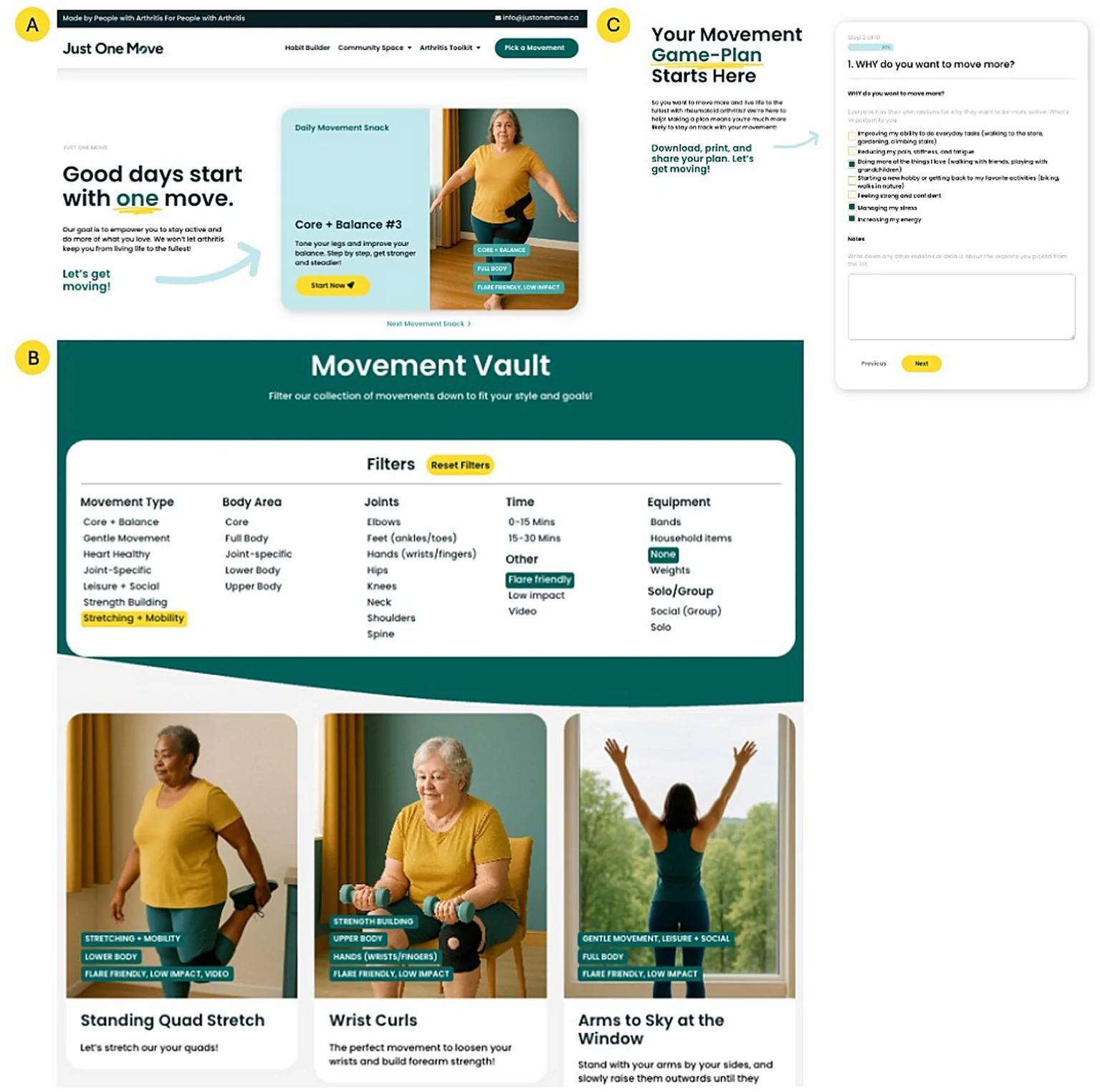
**(A)**: The landing page for Just One Move with a rotating daily movement snack. **(B)**: The Movement Vault with arthritis-friendly movements and customizable filters. **(C)**: Guided action planning with the Habit Builder. Visit JustOneMove.ca to view intervention features in full detail. This figure contains AI-generated individuals. No real photos were used.

**Figure 5:**
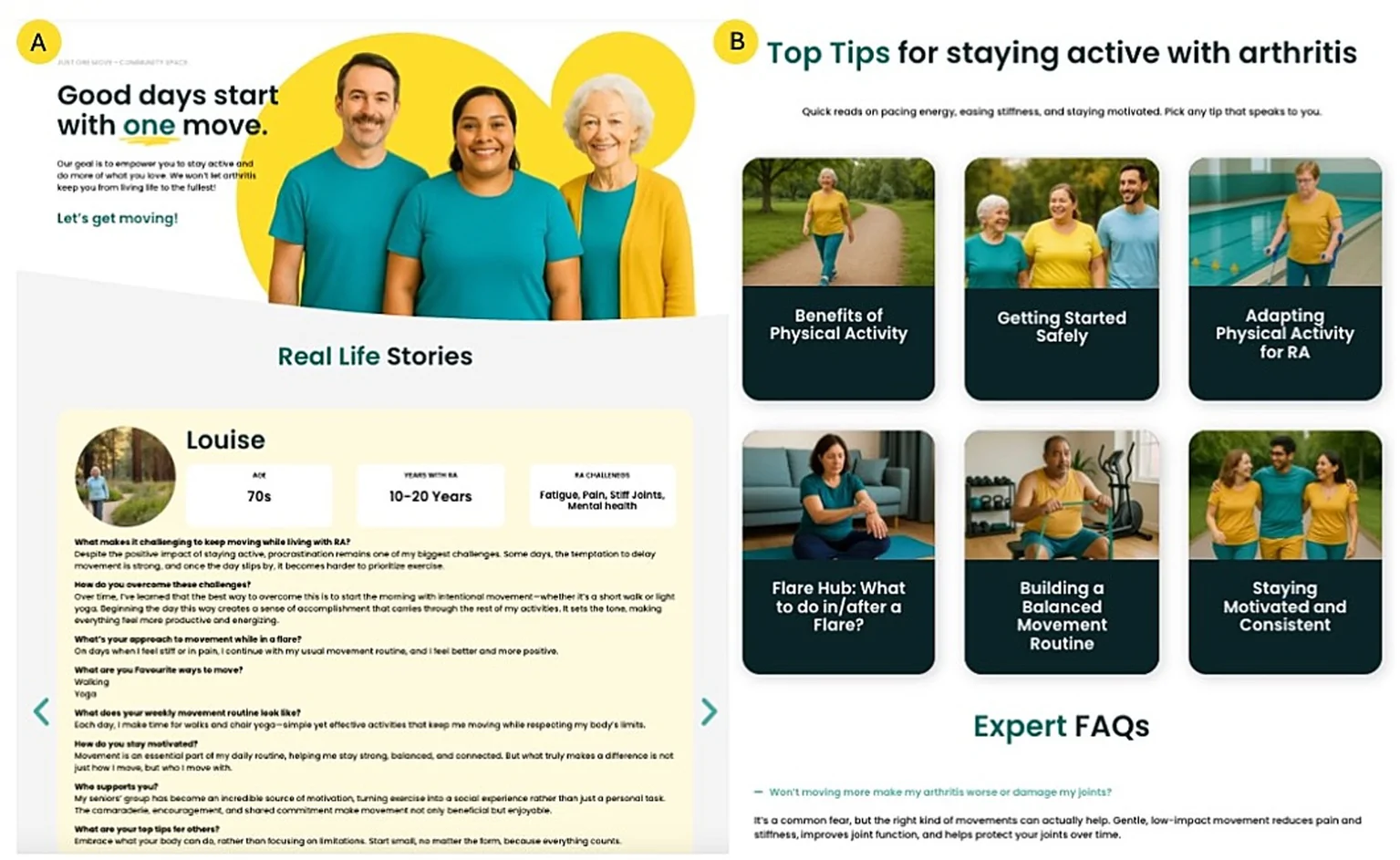
**(A)**: Inspiring real-life stories from others with RA in the Community Space. **(B)**: Expert tips for moving well with RA in the Arthritis Toolkit. Visit JustOneMove.ca to view intervention features in full detail. This figure contains AI-generated individuals. No real photos were used.

On the landing page, users are immediately greeted with our core message, “good days start with one move”, and prompted to start moving with the “daily movement snack”, a short 10–15 min activity with 3–5 movements focusing on 1 or more core areas of physical fitness: cardiovascular, strength, flexibility, balance, combined, social, or gentle movement and rest (Figure 4A). This feature addresses patient partner feedback during the design and refine stages of co-design, which highlighted the need for small, achievable movement options to get started.

In line with Just One Move’s co-defined mission to inspire action, this feature is intended to empower people with RA to move just a little more each day, gradually building physical activity self-efficacy. Self-efficacy was an important physical activity determinant highlighted during our phase 0 preparatory work (9, 12, 27, 28). In addition to the daily movement snack, users are able to “pick a movement” using a green action button in the top right corner of the site.

This takes users to the Movement Vault, a curated selection of arthritis-friendly movements, which can be filtered by movement type, body area, joints involved, duration, equipment required, and other needs including flare-friendly and low impact options (Figure 4B). The Movement Vault directly targets the lack of arthritis-specific exercise recommendations, a well-published physical activity barrier that patient partners reiterated frequently (9). It therefore allows for extensive arthritis-specific tailoring, helping users to find movements that match their needs and fluctuating daily health challenges. Users can save their favorite movements to create a personalized library in “my favourites.” Furthermore, each movement page contains suggestions to try additional related movements or something new in another category, capitalizing on the moment of opportunity to turn one movement into a movement routine.

The next key feature is the Habit Builder, a guided action planning questionnaire that allows users to create a personalized physical activity action plan (Figure 4C). Once completed, users are encouraged to save the plan as a PDF, send it to their email, and share it with others via email or social media. The aim of the habit builder is to transform small movement wins into lasting physical activity habits through goal-setting, action planning, planning for barriers, and leveraging social support for feedback and accountability. While action planning is consistently linked to successful behavior change, patient partners noted the need for an RA-specific tool they could complete on their own.

The Community Space leverages the power of social support, a key component of physical activity behavior change, featuring stories from others with RA as role models that provide inspiration (Figure 5A). The emphasis of the small wins (i.e., a quick share option to celebrate daily movement) and real-life stories (i.e., short stories of the challenges and successes of staying active with RA) is to present a realistic picture of the ups and downs of physical activity while living with RA. Our patient partners emphasized the importance of hearing not just success stories, but also the comfort of knowing that others are facing similar challenges. Users are prompted to submit their own small wins or real-life stories to be an active part of the Just One Move community space.

Patient partners bemoaned that finding reliable, evidence-based, and actionable information on moving well with RA is becoming increasingly difficult. Therefore, the Arthritis Toolkit provides evidence-based, practical expert tips and resources which have been curated by exercise professionals and rheumatology clinicians (Figure 5B). It is intended to improve user knowledge about physical activity for RA and address common questions and concerns. The list of topics covered was directly informed by patient partners based on their lived expertise. For example, specific pages have been developed for helping people get started safely, adapt their physical activity for RA, and understand how to stay active during or after an RA flare. Beyond educating users to increase knowledge, each topic contains a list of related resources for additional support.

Lastly, users can subscribe to the Just One Move monthly newsletter, which contains exclusive subscriber content including a monthly movement challenge emphasizing one domain of physical fitness, a behavior change tip to strengthen movement habits, a featured community member story, and seasonal expert tips for moving well with RA.

### 3.3 Phase 2: mixed methods usability testing survey

#### 3.3.1 Respondent demographics

The 37 survey participants had a median age of 62.0, with 10.0 years living with RA. They were 84% female (*n* = 31/37), 81% White (*n* = 30/37), and 65% physically active (*n* = 24/37). Demographics are summarized in Table 1.

**Table 1:** Demographic characteristics of survey respondents.

Characteristics	Frequency, *n* (%)
Age, Median (IQR)	62.0 (50.8–69.0)
Years with RA, Median (IQR)	10.0 (6.8–21.8)
Gender	
Male	4 (10.8)
Female	31 (83.8)
Non-binary/Gender fluid	1 (2.70)
Not reported	1 (2.70)
Race/Ethnicity	
White/European	30 (81.1)
Southeast Asian	3 (8.11)
Black/African/Caribbean	2 (5.41)
Indigenous (First Nations/Inuit/Metis)	2 (5.41)
Highest level of education	
Some high school	1 (2.70)
High school diploma or equivalent	6 (16.2)
Apprenticeship or trades certificate or diploma	3 (8.11)
College or other non-university certificate or diploma	5 (13.5)
University certificate or diploma	7 (18.9)
Bachelor’s degree	10 (27.0)
Master’s degree	4 (10.8)
Not reported	1 (2.70)
Strenuous exercise (times/week), Median (IQR)	0 (0–2)
Moderate exercise (times/week), Median (IQR)	2 (1–3)
Mild exercise (times/week), Median (IQR)	3 (2–5)
Leisure score index, Median (IQR)	30 (17–39)
Insufficiently active (<14)	6 (16.2)
Moderately active (14–23)	7 (18.9)
Active (>23)	24 (64.9)

IQR, interquartile range.

#### 3.3.2 Website user ratings

Mean website ratings across the 4 MARS quality domains ranged from 3.9/5 ± 0.7 (engagement) to 4.3/5 ± 0.5 (information), with an overall mean quality score of 4.0/5 ± 0.7. Perceived impact scores ranged from 3.6/5 ± 0.9 (Knowledge) to 3.9/5 ± 0.7 (Help seeking behavior), with an overall mean perceived impact of 3.7/5 ± 0.6. The website was rated as 4–5 stars by 73% (*n* = 27/37) of respondents. Of the 24 participants who rated their willingness to pay for the website, 54% (*n* = 13/24) responded “maybe” and 25% (*n* = 6/24) responded “yes.” All ratings are presented in Table 2.

**Table 2:** MARS survey response user ratings.

Domain	Item	Mean (SD)
Engagement	Entertainment	3.8 (0.8)
	Interest	4.2 (0.9)
	Customization	3.4 (1.0)
	Interactivity	3.8 (1.0)
	Target Group	4.4 (1.0)
Overall Engagement		3.9 (0.7)
Functionality	Performance	4.4 (0.8)
	Ease of Use	3.8 (1.1)
	Navigation	3.8 (1.2)
Overall Functionality		4.0 (0.9)
Aesthetics	Layout	4.1 (0.9)
	Graphics	4.4 (0.7)
	Visual Appeal	4.1 (0.6)
Overall Aesthetics		4.2 (0.7)
Information	Quality of Information	4.2 (0.6)
	Quantity of Information	4.4 (0.9)
	Visual Information	4.3 (0.6)
	Credibility of Source	4.4 (0.7)
Overall Information		4.3 (0.5)
Objective Quality		4.0 (0.7)
Quality	Would you recommend this website?	3.9 (1.0)
	How often would you use the site in the next 12 months?	
	0	10.8%
	1–2	8.1%
	3–10	29.7%
	10–50	37.8%
	>50	13.5%
	Would you pay for this website?	
	Yes	25.0%
	Maybe	54.2%
	No	20.8%
	Overall star rating of website	3.9 (0.7)
Subjective Quality		3.6 (1.0)
Perceived Impact	Awareness	3.8 (0.7)
	Knowledge	3.6 (0.9)
	Attitudes	3.6 (0.9)
	Intention to change	3.7 (0.8)
	Help seeking	3.9 (0.7)
	Behavior change	3.8 (0.8)
Overall Perceived Impact		3.7 (0.6)

#### 3.3.3 Preliminary usability testing: qualitative survey data

A summary of the participant perspectives presented in the qualitative data is presented below. Open-ended feedback was provided by 31/37 participants. Raw qualitative data can be found in Supplementary Table 3. Feedback was grouoped into four topics, with topics 1–2 related to the usability of the platform, and topics 3–4 related to its perceived value for people with RA.

#### 3.3.4 Topic 1: just one move is visually appealing and easy to navigate

Participants spoke positively about the usability due to visual appeal and ease of navigation. They liked the attractive colors and graphics, clear headings, and the larger text size broken up into smaller blocks of text for readability. Participant 9 said “The use of clear headings and spacing made it easy to find the information I was looking for without feeling overwhelmed.” Just One Move was easy to navigate for most participants, who appreciated the simple layout and logical flow of content. Participant 22 noted “I liked how clean and easy to navigate the website is. The layout is intuitive, and pages load quickly.”

#### 3.3.5 Topic 2: clearer instructions will make just one move more intuitive for new users

Several participants had challenges navigating the platform without external help. Overall navigation, as well as the purpose of some features, was not immediately intuitive. Participant 27 bemoaned that “It took me quite a while of looking around the site to understand how it works. It was not intuitive for me.” A common suggestion was to provide more details about the purpose of each page and how to use each feature. An onboarding process, with written and video instructions, was presented as a possible solution. Participant 8 indicated “Adding short explainer videos or icons in information-heavy sections would also enhance engagement and understanding.” To improve the Habit Builder, participant 14 mentioned “A clearer explanation on what is happening might be helpful - talk about building the plan.”

Some pages were seen as text-heavy, leaving participants overwhelmed with too much information. Similarly, the real-life stories in the Community Space rotated too fast for participants to read them. This could be improved by slowing down transitions and formatting changes to increase white space, as described by participant 21 “In general, a lot of the text is totally flush with the left side of the monitor. A bit of blank space between the edge of the monitor and the text would make this easier to read.”

#### 3.3.6 Topic 3: just one move inspires confidence with practical tips and support

Participants saw the value of Just One Move for inspiring confidence to move more by making movement approachable, creating a supportive community space, and providing practical tips for overcoming RA-related challenges. They noted it could be especially useful for people who were not active before their RA diagnosis. Participant 4 felt that Just One Move “gives confidence for anyone to take their physical exercise serious no matter the challenges stopping them from exercising.” The Movement Vault and Habit Builder were appreciated for making movement feel approachable through small steps and tips for building long-term habits. The variety of movements allowed participants to find what works for them each day. Participant 30 liked “The choice between different movement lengths - Single Move, 3-5 moves, or Many Moves. Can therefore vary it each day.” Including video and written instructions was seen as important to encourage practice new movements. The community space and arthritis toolkit were described as valuable pages for gaining motivation and learning practical tips to build and maintain activities while navigating the ups and downs of RA. Participant 10 said “I really love how this website allows me to share my little wins and see other people’s stories. It’s so inspiring! The tips and motivation available here keep me going and push me to get better physically every day.”

#### 3.3.7 Topic 4: users want options for customization and more trusted content to choose from

Not all participants found Just One Move valuable. One participant noted that “Because I am already physically active and reasonably fit, and access a different exercise regime, it didn’t really increase my knowledge or motivation.” Furthermore, one participant was not interested in the real-life stories shared by others with RA. To make Just One Move more useful, participants wanted more flexibility to customize Just One Move to their needs and receive personalized advice, an expanded library of RA-friendly movements, and trusted content from exercise professionals. For example, participant 22 said “I think having certified coaches or health professionals share weekly tips or Q&As could add more value and trust.”

One participant did not like anything about Just One Move, without elaborating on why this was the case. Five participants commented that they saw nothing to dislike and no needs for improvement.

## 4 Discussion

### 4.1 Summary of findings

Together with a core team of people with RA, researchers, and design experts, the Just One Move intervention was co-designed using a 4-step, user-centered process. Just One Move delivers interactive features that match user needs and preferences, leveraging evidence-based intervention strategies and BCTs. Preliminary feedback indicates that people with RA see Just One Move as a high-quality, user-friendly tool that motivates them towards physical activity behavior change.

### 4.2 Comparison with prior work

#### 4.2.1 The co-design process

Trust and team cohesion developed through partner engagement during the preparation phase provided an ideal starting point for the co-design process. Where the co-development of similar interventions reported challenges balancing research knowledge and target user requests, we blended these dimensions based on a strong mutual understanding between researchers and patient partners (42). To prioritize user needs, intervention design was firmly grounded in the priorities of people with RA, which were linked to evidence-based strategies. Similar to related studies, difficulties arose when refining feature requests based on available time and resources (43–45). This challenge may be unavoidable given increasing resource limitations in research and healthcare, yet clear expectations and communication can reduce tensions between different interest holders. Interestingly, our co-design was shorter than most prior studies, lasting only 7 months. While there is no ideal timeline for co-design, the present study indicates that meaningful collaboration is still possible in short-term projects. A well-defined goal and strong preparatory work with co-design partners helped facilitate efficient and effective execution. However, our process still required high-intensity engagement, with weekly meetings, and complex coordination to engage partners with varied schedules and feedback preferences.

#### 4.2.2 Co-design successes and challenges

Trusting relationships with partners, including people with RA, were established during the preparation phase and introductory meetings that strengthened personal connection. Long-term involvement led to a mutual appreciation of the need to blend behavioral science evidence with the needs and preferences of people with RA to create an impactful intervention. Partners appreciated flexible engagement options, allowing them to opt-in to each phase of the process, provide asynchronous feedback via email, and attend evening meetings. However, accommodating varied schedules and integrating feedback from emails and meetings requires additional resources and planning. Design sprint methods led by a design professional facilitated creative thinking and amplified quieter voices on the co-design team. Despite this, moving from creative big picture ideas to well-defined concepts required difficult decisions, balancing between incorporating diverse ideas and maintaining feasibility within timeline and resource constraints. Defining clear expectations and explaining all decisions to partners were key strategies to maintain trust during co-design.

#### 4.2.3 The just one move intervention

Just One Move applied user-centered design with behavioral science to address key gaps in digital physical activity resources for people with RA. As shown in our environmental scan, existing resources in this space rated low for actionability, using BCTs aimed primarily at increasing knowledge (23). Information alone is not sufficient to change behavior. Therefore, Just One Move integrated action-oriented BCTs including goal setting and action planning, which increase self-efficacy to move from intention to action (46–48). All intervention components (e.g., the movement vault) of Just One Move targeted high priority barriers (e.g., lack of RA-specific tailoring of physical activity advice) and facilitators identified by people with RA (12). Planning for barriers, or coping planning, is an important BCT for maintaining physical activity habits long-term (49).

The intervention shares components and BCTs with a co-designed supervised physical activity intervention for RA and a digital platform for people with Osteoarthritis (43, 45). For example, common components were social support and action planning, designed to gradually increase self-efficacy. Interestingly, each co-design process led to different user-friendly approaches to deliver these BCTs, highlighting the value of user-centered design to tailor interventions. Live interaction with peers and exercise professionals, a common component of RA-specific supervised programs, was frequently requested by our patient partners (50). It may be possible to provide synchronous support within Just One Move via telehealth with additional resources. However, these elements add significant complexity: training of interventionists, coordinating of sessions, and fidelity monitoring.

#### 4.2.4 Preliminary usability testing

Preliminary feedback indicated that people with RA see Just One Move as a user-friendly and potentially impactful digital intervention for helping them become and stay physically active. MARS ratings were significantly higher than 6 existing physical activity apps for people with RA (51). Differences were especially pronounced for engagement, information, and subjective quality. This holds true when comparing to broader digital physical activity interventions and rheumatology self-management interventions (52, 53). Given the value of user perceptions for increasing digital intervention engagement and effectiveness, above average MARS ratings speak to the potential impact of Just One Move (54–56). Prior digital tools rarely used co-design approaches and evidence-based strategies to strengthen interventions (51, 52). Therefore, the higher ratings for Just One Move may reflect the positive impact of our evidence-based, user-centered co-design process. Previous research found that, across 6 RA-specific physical activity apps, those featuring a greater number of BCTs had higher MARS quality ratings (51). Caution must be taken in comparing ratings across studies, however, given the inherent subjectivity in MARS scoring.

Our qualitative data provided valuable insights into what shapes perceptions of digital intervention usability and usefulness for people with RA. Visual appeal and easy navigation are two core principles for digital design that people with RA appreciated about Just One Move (55–57). However, the lack of clear instructions to explain functionality was a challenge. This is a common pitfall for unsupervised physical activity interventions, which require well-defined onboarding pathways to ensure engagement as intended by developers (58, 59). Key contributors to perceived usefulness, including the Movement Vault and Community Space, echo past research on the importance of offering personal choice (i.e., autonomy) and providing social support in physical activity interventions (60, 61). Social support may be especially valuable for people with RA and other chronic conditions, where group-based interventions promote a strong sense of belonging (39, 62, 63). Despite our usability testing results, positive perceptions alone do not guarantee that Just One Move will prompt sustained physical activity behavior change.

### 4.3 Implications for research and practice

Further research is needed to determine the effectiveness of Just One Move to increase physical activity levels and improve key health outcomes for people with RA. First, the intervention will be revised based on user feedback, adding onboarding tips and more customizable movement content. The potential for enhancing support via remote health coaching and peer mentorship will be explored. Both features were highly requested by users and can effectively increase physical activity, though significant resources are required to develop and deliver these intervention components (64–66). Additional evaluation with more diverse RA population is also warranted, addressing sampling biases seen herein. Next, definitive randomized trials will be required to test for feasibility, effects on health behaviors and clinical outcomes, and inform implementation into RA care. If Just One Move is shown to be effective to increase physical activity and improve relevant health outcomes (e.g., function, fatigue), it can be integrated alongside other self-care interventions in rheumatology care pathways. Rheumatologists can refer to these interventions alongside continued medical treatment. Strategic implementation science-based approaches will be required to facilitate adoption, integration into clinical teams, pathways, and systems, and maintain implementation. Our team is currently working with rheumatology healthcare providers to co-design toolkits that improve physical activity promotion practices in clinical care (28). A detailed discussion on implementation opportunities and challenges for physical activity support in rheumatology care has been published to guide meaningful implementation (67). Ultimately, improved implementation of evidence-based physical activity support in rheumatology care has the potential to improve individual well-being and population health outcomes.

The co-design process outlined herein may also be adapted to develop tailored physical activity support for other chronic conditions. Practical challenges remain to balance iterative intervention refinement and meaningful engagement with the realities of limited budgets and timelines for completing co-design work. Using time-efficient processes from software development (e.g., design sprints) alongside the United Kingdom MRC/NIHR guidelines and behavioral science theories may enable timely, evidence-based intervention co-design. Furthermore, using the PEIRS-22 or other validated measures for interim process evaluations can foster continuous improvement of partner engagement. Additional research is needed to measure the actual downstream impact of applying co-design to intervention development, which remain difficult to capture.

### 4.4 Strengths and limitations

The study is not without its limitations. A strength is the rigorous co-design process that integrated user needs and priorities, research evidence, behavioral science theories and frameworks, and design expertise. Despite purposive recruitment to gather diverse perspectives, participation from underrepresented groups was limited across both phases. More work is needed to test and tailor Just One Move for diverse populations. Usability testing results must be interpreted with caution due to the small, relatively heterogenous participant sample and cross-sectional nature. As survey respondents only used Just One Move briefly, perceptions on usability and usefulness may change with long-term use. This is being explored during an ongoing 8-week feasibility study. Understanding the impact of Just One Move on physical activity levels and other health outcomes will require rigorous trials.

### 4.5 Conclusion

We co-developed Just One Move, a promising RA-specific digital physical activity intervention alongside people with RA, rheumatology providers, and exercise behavior change experts. The intervention was grounded in the needs and preferences of people with RA, providing behavior change support that blends proven behavioral science strategies and user-centered design principles. While Just One Move was perceived as a promising platform for increasing physical activity among users, rigorous evaluation is needed to understand its effectiveness and guide implementation into rheumatology care. Elements from the iterative co-design process and resultant intervention can inspire the development of user-friendly, evidence-based digital behavior change interventions for people living with chronic conditions.

## Data Availability

The raw data supporting the conclusions of this article will be made available by the authors, without undue reservation.

## References

[1] Vos T Lim SS Abbafati C Abbas KM Abbasi M Abbasifard M . Global burden of 369 diseases and injuries in 204 countries and territories, 1990–2019: a systematic analysis for the global burden of disease study 2019. Lancet. (2020) 396:1204–22. doi: 10.1016/S0140-6736(20)30925-9, 33069326 PMC7567026

[2] Gullick NJ Scott DL. Co-morbidities in established rheumatoid arthritis. Best Pract Res Clin Rheumatol. (2011) 25:469–83. doi: 10.1016/j.berh.2011.10.009, 22137918

[3] Madsen SG Danneskiold-Samsøe B Stockmarr A Bartels EM. Correlations between fatigue and disease duration, disease activity, and pain in patients with rheumatoid arthritis: a systematic review. Scand J Rheumatol. (2016) 45:255–61. doi: 10.3109/03009742.2015.1095943, 26690505

[4] Brady SM Veldhuijzen van Zanten J Dinas PC . Effects of lifestyle physical activity and sedentary behaviour interventions on disease activity and patient- and clinician- important health outcomes in rheumatoid arthritis: a systematic review with meta-analysis. BMC Rheumatol. (2023) 7:27. doi: 10.1186/s41927-023-00352-9 PMC10481589 37674187

[5] Osthoff AK Juhl CB Knittle K Dagfinrud H Hurkmans E Braun J . Effects of exercise and physical activity promotion: meta-analysis informing the 2018 EULAR recommendations for physical activity in people with rheumatoid arthritis, spondyloarthritis and hip/knee osteoarthritis. RMD Open. (2018) 4:e000713. doi: 10.1136/rmdopen-2018-000713 PMC6307596 30622734

[6] Santos AC Willumsen J Meheus F Ilbawi A Bull FC. The cost of inaction on physical inactivity to public health-care systems: a population-attributable fraction analysis. Lancet Glob Health. (2023) 11:e32–9. doi: 10.1016/S2214-109X(22)00464-8, 36480931 PMC9748301

[7] Osthoff AK Niedermann K Braun J Adams J Brodin N Dagfinrud H . 2018 EULAR recommendations for physical activity in people with inflammatory arthritis and osteoarthritis. Ann Rheum Dis. (2018) 77:1251–60. doi: 10.1136/annrheumdis-2018-213585, 29997112

[8] England BR Smith BJ Baker NA Barton JL Oatis CA Guyatt G . 2022 American College of Rheumatology guideline for exercise, rehabilitation, diet, and additional integrative interventions for rheumatoid arthritis. Arthritis Rheumatol. (2023) 75:1299–311. doi: 10.1002/acr.25117 PMC10947582 37227071

[9] Ester M Gillespie D Zafar S Githumbi R Dhiman K Adhikari K . Changing physical activity behavior among individuals with inflammatory arthritis: an umbrella review of current behaviors, behavioral determinants, and behavior change interventions. BMC Rheumatol. (2025) 10:3. doi: 10.1186/s41927-025-00601-z PMC12781547 41340157

[10] BARFOT Study Group Malm K Bergman S Bremander A Larsson I Andersson ML. Discussions of lifestyle habits as an integral part of care management: a cross-sectional cohort study in patients with established rheumatoid arthritis in Sweden. Rheumatol Adv Pract. (2019) 3:rkz039 10.1093/rap/rkz039 PMC6827554 31701084

[11] Freid LM Ogdie A Baker JF. Physical activity patterns in people with inflammatory arthritis indicate they have not received recommendation-based guidance from health care providers. ACR Open Rheumatology. (2020) 2:582–7. doi: 10.1002/acr2.11183, 32985797 PMC7571386

[12] Ester M Dhiman K Githumbi R Sipley M Dahal KA Barber CE. Priority setting of physical activity barriers and facilitators among individuals with rheumatoid arthritis: a nominal group technique study. J Rheumatol. (2025) 52:979–87. doi: 10.3899/jrheum.2025-0191, 40713172

[13] Veldhuijzen van Zanten JJ Rouse PC Hale ED Ntoumanis N Metsios GS Duda JL . Perceived barriers, facilitators and benefits for regular physical activity and exercise in patients with rheumatoid arthritis: a review of the literature. Sports Med. (2015) 45:1401–12. doi: 10.1007/s40279-015-0363-2, 26219268 PMC4579262

[14] Tran M Pesah E Turk-Adawi K Supervia M Jimenez FL Oh P . Cardiac rehabilitation availability and delivery in Canada: how does it compare with other high-income countries? Can J Cardiol. (2018) 34:S252–62. doi: 10.1016/j.cjca.2018.07.413, 30274636

[15] Fowles JR Shields C d’Entremont L McQuaid S Barron B Dunbar P. Implementation of resources to support patient physical activity through diabetes centres in Nova Scotia: the effectiveness of enhanced support for exercise participation. Can J Diabetes. (2014) 38:423–31. doi: 10.1016/j.jcjd.2014.02.008, 25065476

[16] Roos EM Grønne DT Skou ST Zywiel M McGlasson R Barton CJ . Outcomes following the glad program for patients with symptomatic knee and hip osteoarthritis in Denmark, Canada and Australia. A longitudinal analysis including 28,370 patients. Osteoarthr Cartil. (2020) 28:S31–2. doi: 10.1016/j.joca.2020.02.051 33561542

[17] Wu B Zhang M Hu H Wu X. The effectiveness of telemedicine in patients with rheumatoid arthritis: an overview of systematic reviews and Meta-analysis. Telemed J E Health. (2023) 29:1747–58. doi: 10.1089/tmj.2023.0074, 37074335

[18] Bearne LM. Physical activity in rheumatoid arthritis—is it time to push the pace of change? Rheumatol Adv Pract. (2023) 7:rkac107. doi: 10.1093/rap/rkac107, 36699548 PMC9870704

[19] Demmelmaier I Iversen MD. How are behavioral theories used in interventions to promote physical activity in rheumatoid arthritis? A systematic review. Arthritis Care Res. (2018) 70:185–96. doi: 10.1002/acr.23265, 28437600

[20] Salwen-Deremer JK Khan AS Martin SS Holloway BM Coughlin JW. Incorporating health behavior theory into mHealth: an examination of weight loss, dietary, and physical activity interventions. J Technol Behav Sci. (2020) 5:51–60. doi: 10.1007/s41347-019-00118-6

[21] Skivington K Matthews L Simpson SA Craig P Baird J Blazeby JM . A new framework for developing and evaluating complex interventions: update of Medical Research Council guidance. BMJ. (2021) 374:n2061. doi: 10.1136/bmj.n2061, 34593508 PMC8482308

[22] Wemyss D Lobsiger-Kägi E Jud S Cellina F. Leveraging realities of saving energy at home: contributions of co-design to behavioural interventions. Energy Res Soc Sci. (2023) 104:103258. doi: 10.1016/j.erss.2023.103258

[23] Vargas C Whelan J Brimblecombe J Allender S. Co-creation, co-design, co-production for public health–a perspective on definitions and distinctions. Public Health Research and Practice. (2022) 32:e3222211. doi: 10.17061/phrp3222211, 35702744

[24] Vargas C Longworth GR Zorbas C Needham C Ugalde A Allender S. Establishing fundamental co-design principles and strategies for public health research practice. CoDesign. (2026) 1-22:1–22. doi: 10.1080/15710882.2026.2626736, 37339054

[25] Nierse CJ Schipper K Van Zadelhoff E Van De Griendt J Abma TA. Collaboration and co-ownership in research: dynamics and dialogues between patient research partners and professional researchers in a research team. Health Expect. (2012) 15:242–54. doi: 10.1111/j.1369-7625.2011.00661.x, 21332617 PMC5060620

[26] Appleby B Cowdell F Booth A. Knowledge mobilization in bridging patient-practitioner-researcher boundaries: a systematic integrative review. J Adv Nurs. (2021) 77:523–36. doi: 10.1111/jan.14586 33068022

[27] Ester M Zafar S Dhiman K Graveline C McKinnon A Hoens A . Online physical activity resources for individuals with rheumatoid arthritis: an environmental scan and quality appraisal. BMJ Open. (2025) 15:e094220. doi: 10.1136/bmjopen-2024-094220, 39979059 PMC11843015

[28] Ester M Gillespie DC Dhiman K Githumbi R Sipley M Adhikari K . Priority setting of healthcare provider barriers and facilitators to supporting patient physical activity in rheumatology: a nominal group technique study. BMJ Open Sport Exerc Med. (2025) 11:2669. doi: 10.1136/bmjsem-2025-002669, 41090105 PMC12517023

[29] Hamilton CB Hoens AM Backman CL English K McKinnon AM McQuitty S Workbook to guide the Development of a patient Engagement In Research (PEIR) Plan Arthritis Research Canada (2023) Available online at: https://www.arthritisresearch.ca/wp-content/uploads/2018/06/PEIR-Plan-Guide.pdf (Accessed January 31, 2026).

[30] Still B Crane K. Fundamentals of user-Centered design: A Practical Approach. Boca Raton, Florida: CRC press (2017).

[31] Michie S. Atkins L. West R. The Behaviour change wheel: a guide to Designing Interventions. Surrey: Silverback Publishing. (2014).

[32] Michie S Richardson M Johnston M Abraham C Francis J Hardeman W . The behavior change technique taxonomy (v1) of 93 hierarchically clustered techniques: building an international consensus for the reporting of behavior change interventions. Ann Behav Med. (2013) 46:81–95. doi: 10.1007/s12160-013-9486-6, 23512568

[33] Tricco AC Zarin W Clement F Abou-Setta AM Curran JA LeBlanc A . Introducing the strategy for patient oriented research (SPOR) evidence Alliance: a partnership between researchers, patients and health system decision-makers to support rapid-learning and responsive health systems in Canada and beyond. Facets. (2022) 7:639–53. doi: 10.1139/facets-2021-0127, 34819996

[34] Hamilton CB Hoens AM McQuitty S McKinnon AM English K Backman CL . Development and pre-testing of the patient engagement in research scale (PEIRS) to assess the quality of engagement from a patient perspective. PLoS One. (2018) 13:e0206588–18. doi: 10.1371/journal.pone.0206588, 30383823 PMC6211727

[35] Hamilton CB Leese JC Hoens AM Li LC. Framework for advancing the reporting of patient engagement in rheumatology research projects. Curr Rheumatol Rep. (2017) 19:38. doi: 10.1007/s11926-017-0666-4, 28631065 PMC5750042

[36] Wangsa K Chugh R Karim S Sandu R. A comparative study between design thinking, agile, and design sprint methodologies. Int J Agile Syst Manag. (2022) 15:225–42. doi: 10.1504/ijasm.2022.124916

[37] Godin G Shephard RJ. Leisure time exercise questionnaire. Can J Appl Sport Sci. (1997) 4:18–22.

[38] Bateman A Myers ND Chen S Lee S. Measurement of physical activity self-efficacy in physical activity-promoting interventions in adults: a systematic review. Meas Phys Educ Exerc Sci. (2022) 26:141–54. doi: 10.1080/1091367x.2021.1962324

[39] Stoyanov SR Hides L Kavanagh DJ Wilson H. Development and validation of the user version of the Mobile application rating scale (uMARS). JMIR Mhealth Uhealth. (2016) 4:e72. doi: 10.2196/mhealth.5849, 27287964 PMC4920963

[40] Elo S Kyngäs H. The qualitative content analysis process. J Adv Nurs. (2008) 62:107–15. doi: 10.1111/j.1365-2648.2007.04569.x, 18352969

[41] Johnston M Carey RN Connell Bohlen LE Johnston DW Rothman AJ De Bruin M . Development of an online tool for linking behavior change techniques and mechanisms of action based on triangulation of findings from literature synthesis and expert consensus. Transl Behav Med. (2021) 11:1049–65. doi: 10.1093/tbm/ibaa050, 32749460 PMC8158171

[42] Creaser AV Bingham DD Bennett HA Costa S Clemes SA. The development of a family-based wearable intervention using behaviour change and co-design approaches: move and connect. Public Health. (2023) 217:54–64. doi: 10.1016/j.puhe.2023.01.018, 36854251

[43] Salmon VE Hewlett S Walsh NE Kirwan JR Morris M Urban M . Developing a group intervention to manage fatigue in rheumatoid arthritis through modifying physical activity. BMC Musculoskelet Disord. (2019) 20:194. doi: 10.1186/s12891-019-2558-4, 31054567 PMC6500086

[44] Revenäs Å Opava CH Martin C Demmelmaier I Keller C Åsenlöf P. Development of a web-based and mobile app to support physical activity in individuals with rheumatoid arthritis: results from the second step of a co-design process. JMIR Res Protoc. (2015) 4:e22. doi: 10.2196/resprot.3795, 25665589 PMC4342685

[45] Berry A McCabe CS Muir S Walsh N. Systematic co-development and testing of a digital behaviour change intervention for osteoarthritis and physical activity: theoretical mapping and acceptability study. Digit Health. (2023) 9:20552076231204425. doi: 10.1177/20552076231204425, 37808237 PMC10559706

[46] Howlett N Trivedi D Troop NA Chater AM. Are physical activity interventions for healthy inactive adults effective in promoting behavior change and maintenance, and which behavior change techniques are effective? A systematic review and meta-analysis. Transl Behav Med. (2019) 9:147–57. doi: 10.1093/tbm/iby010, 29506209 PMC6305562

[47] Samdal GB Eide GE Barth T Williams G Meland E. Effective behaviour change techniques for physical activity and healthy eating in overweight and obese adults; systematic review and meta-regression analyses. Int J Behav Nutr Phys Act. (2017) 14:42. doi: 10.1186/s12966-017-0494-y, 28351367 PMC5370453

[48] Williams SL French DP. What are the most effective intervention techniques for changing physical activity self-efficacy and physical activity behaviour—and are they the same? Health Educ Res. (2011) 26:308–22. doi: 10.1093/her/cyr005, 21321008

[49] Mattila E Horgan G Palmeira AL O'Driscoll R Stubbs RJ Heitmann BL . Evaluation of the immediate effects of web-based intervention modules for goals, planning, and coping planning on physical activity: secondary analysis of a randomized controlled trial on weight loss maintenance. J Med Internet Res. (2022) 24:e35614. doi: 10.2196/35614, 35436232 PMC9052022

[50] Salmon VE Hewlett S Walsh NE Kirwan JR Cramp F. Physical activity interventions for fatigue in rheumatoid arthritis: a systematic review. Phys Ther Rev. (2017) 22:12–22. doi: 10.1080/10833196.2016.1277454

[51] Bearne LM Sekhon M Grainger R La A Shamali M Amirova A . Smartphone apps targeting physical activity in people with rheumatoid arthritis: systematic quality appraisal and content analysis. JMIR Mhealth Uhealth. (2020) 8:e18495. doi: 10.2196/18495, 32706727 PMC7404016

[52] Knitza J Tascilar K Messner EM Meyer M Vossen D Pulla A . German mobile apps in rheumatology: review and analysis using the mobile application rating scale (MARS). JMIR Mhealth Uhealth. (2019) 7:e14991. doi: 10.2196/14991, 31381501 PMC6699116

[53] Paganini S Terhorst Y Sander LB Catic S Balci S Küchler AM . Quality of physical activity apps: systematic search in app stores and content analysis. JMIR Mhealth Uhealth. (2021) 9:e22587. doi: 10.2196/22587, 34106073 PMC8262667

[54] Grady A Pearson N Lamont H Leigh L Wolfenden L Barnes C . The effectiveness of strategies to improve user engagement with digital health interventions targeting nutrition, physical activity, and overweight and obesity: systematic review and meta-analysis. J Med Internet Res. (2023) 25:e47987. doi: 10.2196/47987, 38113062 PMC10762625

[55] Hentati A Forsell E Ljótsson B Kaldo V Lindefors N Kraepelien M. The effect of user interface on treatment engagement in a self-guided digital problem-solving intervention: a randomized controlled trial. Internet Interv. (2021) 26:100448. doi: 10.1016/j.invent.2021.100448, 34471610 PMC8387893

[56] Boucher EM Raiker JS. Engagement and retention in digital mental health interventions: a narrative review. BMC Digit Health. (2024) 2:52. doi: 10.1186/s44247-024-00105-9

[57] Denison-Day JL Muir S Newell C Appleton KM. The role of aesthetics in intentions to use digital health interventions. PLOS digital health. (2023) 2:e0000274. doi: 10.1371/journal.pdig.0000274, 37347727 PMC10286978

[58] Bannell DJ France-Ratcliffe M Buckley BJ Crozier A Davies AP Hesketh KL . Adherence to unsupervised exercise in sedentary individuals: a randomised feasibility trial of two mobile health interventions. Digit Health. (2023) 9:20552076231183552. doi: 10.1177/20552076231183552, 37426588 PMC10328121

[59] Chaterjee A Gerdes M Prinz A Martinez S. Realizing the effectiveness of digital interventions on sedentary behavior (physical inactivity, unhealthy habit, improper diet) monitoring and prevention approaches as a meta-analysis. J Med Internet Res. (2021) 23:e26251. doi: 10.2196/26931

[60] Pollet S Denison-Day J Bradbury K Essery R Grey E Western M . A qualitative exploration of perceptions of a digital intervention to promote physical activity in older adults. J Aging Phys Act. (2020) 29:442–54. doi: 10.1123/japa.2019-0484, 33348316

[61] Craig BA Morrison L McDonough MH Sabiston CM Bennett E Doré I . Social support in physical activity interventions for adults: an overview of reviews. Kinesiol Rev. (2025) 14:277–89. doi: 10.1123/kr.2024-0026

[62] McDonough MH Beselt LJ Daun JT Shank J Culos-Reed SN Kronlund LJ . The role of social support in physical activity for cancer survivors: a systematic review. Psycho-Oncology. (2019) 28:1945–58. doi: 10.1002/pon.5171, 31278800

[63] Withall J Haase AM Walsh NE Young A Cramp F. Physical activity engagement in early rheumatoid arthritis: a qualitative study to inform intervention development. Physiotherapy. (2016) 102:264–71. doi: 10.1016/j.physio.2015.07.002, 26386844

[64] Sezgin MG Bektas H. The effect of coaching programs on physical activity and pain in patients with rheumatoid arthritis: a systematic review and meta-analysis of randomized controlled trials. Pain Manag Nurs. (2023) 24:549–57. doi: 10.1016/j.pmn.2023.06.009, 37543452

[65] Karp N Yazdany J Schmajuk G. Peer support in rheumatic diseases: a narrative literature review. Patient Prefer Adherence. (2023) 17:2433–49. doi: 10.2147/PPA.S391396, 37808273 PMC10557966

[66] Sezgin MG Bektas H. Effect of peer mentoring on physical activity in patients with cancer: a systematic review and meta-analysis of randomised controlled trials. J Clin Nurs. (2023) 32:2410–8. doi: 10.1111/jocn.16320, 35396757

[67] Metsios GS Kitas GD. Physical activity, exercise and rheumatoid arthritis: effectiveness, mechanisms and implementation. Best Pract Res Clin Rheumatol. (2018) 32:669–82. doi: 10.1016/j.berh.2019.03.013, 31203925

