# Supplementary material for: Just One Move: co-design and usability testing of a digital physical activity behavior change platform for people with rheumatoid arthritis
Source: Front Public Health. 2026 Oct 29;14:1972848. doi: 10.3389/fpubh.2026.1972848 (PMC13755205; doi:10.3389/fpubh.2026.1972848)
Supplement: Supplementary file 1 [file Data_Sheet_1.DOCX]

Supplementary Material

*Just One Move: Co-developing and evaluating a digital physical activity behavior change platform for people with rheumatoid arthritis*

# 1 Supplementary Data

## 1.1 Supplementary File 1: Design sprint facilitation agenda for step 2 of the co-design process

**Objectives**

The goal of this session is to explore solutions for those who are faced with Rheumatoid Arthritis, prioritize the ideas, and generate next steps for testing and validation.

**Agenda**

| **RUN TIME** | **ACTIVITY** | **DESCRIPTION** |
| --- | --- | --- |
| 000 | Opening remarks + Welcome | • Setting the stage, welcome and introductions |
| 0005 | Ice Breaker | • Let’s get to know who is in the room and get introduced to the design process |
| 0015 | Card Sorting | • Prioritize our ideas for the future |
| 0040 | How Might We | • Questions to fuel creative confidence |
| 0100 | Analogies | • Where have we seen these challenges before? |
| 0115 | The Big Storm | • Raining Ideas |
| 0130 | Prioritization | • Which ideas would make the biggest splash? |
| 0145 | Closing + Next Steps | • Where do we go from here? |

## 1.2 Supplementary File 2: Guide for user testing conducted during step 3 of the co-design process.

**User Testing Guide: Rheumatoid Arthritis Physical Activity Website Prototype**

Joint Moves/Move Well RA/RA in Motion/Move-to-Thrive RA

**Introduction**

Thank you for participating in this user testing session. Your feedback is valuable in helping us refine our website to better support individuals with rheumatoid arthritis in staying active. This session will involve exploring the website, completing specific tasks, and sharing your thoughts on your experience.

**Duration:** 45-60 minutes
**Goal:** Assess usability, navigation, clarity, and overall user experience.

**Part 1: Activity Scenarios (30-40 min)**

Participants will be given tasks to complete while thinking aloud. Encourage them to express their thoughts, expectations, and frustrations.

**Activity 1: Explore and Navigate the Website**

**Scenario:** You’ve just landed on the homepage of this website. Take a moment to explore and describe your initial impressions.

- What stands out to you?
- What do you expect to find here?
- How easy is it to understand what the site offers?

**Follow-up prompts:**

- Was there anything confusing or unclear?
- What would you do next if you were using this site for the first time?
- Go ahead and take a few minutes to delve deeper into the website, focusing on what would be most relevant or interesting for you.
  - Talk us through your thoughts as you explore.

**Notes**

**Activity 2: Pick an activity for today**

**Scenario:** You’re looking for an activity that you can do right now.

- Why did you go to “RA friendly workouts”? (Or: “activity of the day”)
  - What would help you make a quick decision and get moving?
- What level of detail would you want for each activity?
  - What info is essential and what info could be left for you to decide?

**Follow-up prompts:**

- What additional details would you like to see?
- What would make you likely to follow an activity from this website?

**Notes**

**Activity 3: Set an Activity Goal and Plan**

**Scenario:** You want to set a personal activity goal and create a plan to stay active.

- Can you find where to set an activity goal?
- Walk me through how you would create your plan.

**Follow-up prompts:**

- What information would you be willing to provide to help with the action planning and goal setting?
- What would be a useful format for the final activity plan?
  - Would you want to print it?
  - What information would you want it to include?
- What would make this feature more useful?

**Notes**

**Activity 4: Engage with the Community Section**

**Scenario:** You’re looking for motivation and support from others with similar experiences.

- Where would you go to find success stories or community discussions?
- What type of content would you want to find in the community section of the site?

**Follow-up prompts:**

- How comfortable would you feel engaging with the community here?
- Were the differences between the pages clear?
  - What was unclear?
  - What changes could we make?
- What would encourage you to return and engage regularly?

**Notes**

**Activity 5: Learn about safety and suitable movement for you**

**Scenario:** You’re unsure what activity would be safe and suitable for someone with rheumatoid arthritis, so you want to learn more.

- Where would you go to find expert advice or research-based information?
- What types of information would be the most important to see here at first glance, without feeling overwhelming?

**Follow-up prompts:**

- Was would you want to learn about the most?
- What questions might remain unanswered?

**Notes**

**Part 2: Post-Test Questions (5-10 min)**

These questions help gather final impressions and suggestions.

1. Overall, how was your experience using the website?
   a. What did you like most about the site?
   b. What did you find frustrating or confusing?
2. If you could change one thing about this website, what would it be?
3. How likely are you to return to this website in the future? Why or why not?
4. Do you have any additional feedback or suggestions?

**Notes**

**Conclusion**

Thank you for your time and feedback! Your insights will help improve this website to better support individuals with RA in staying active. If you have any additional thoughts after this session, feel free to share them with us.

# 2 Supplementary Tables

## 2.1 Supplementary Table 1: Complete details about partner engagement throughout the study.

| Who: Type of affiliation | Individuals  Research team, working group |
| --- | --- |
| Who: PRP characteristics | PRP category: patients  Number of PRPs: 14 adults living with RA from across Canada |
| How: Initiation of Engagement | 3 Jointly initiated |
| How: Methods of Contribution | 1 Complete interview/survey  2 Participate in discussions  3 Participate in research-related tasks  5 Participate virtually |
| How: Level of Engagement | Level 3 Collaborated |
| When: Stages of Research Cycle | Preparatory Phase  **Step 1 Identifying and Prioritising**  Execution Phase  **Step 4 Undertaking (Recruiting**  **Participants, Collecting Data,**  **Analyzing Data, Interpreting**  **Data)**  Translation Phase  **Step 5 Disseminating**  **Step 6 Implementing** |

Supplementary Table 1: Complete details about partner engagement throughout the study

## 2.3 Supplementary Table 2: Summary of meeting frequency and duration during the co-design phase

| Title | Research Team | Patient Partners | External: Design Team, Web Developer | Details | Duration | Sessions | Total Time |
| --- | --- | --- | --- | --- | --- | --- | --- |
| Patient Partner Kick Off | 4 | 14 | 0 | Project launch meeting with patient partners (2 sessions: Jul 18, 23) | 60 | 2 | 120 |
| Nominal Group Technique sessions | 5 | 14 | 0 | Priority setting: 3 NGT sessions, 5 attendees each (4 at last session) | 60 | 3 | 180 |
| Co-design Kick Off | 3 | 10 | 0 | 2 meetings (Oct 31, Nov 6) to summarize findings to date and discuss co-design next steps, gathered early input from patient partners | 60 | 2 | 120 |
| Design Sprint Sessions | 4 | 11 | 1 | Facilitated by Josh/AHS Design Lab, 2 sessions Jan 21, Jan 22 (5 and 6 participants) | 90 | 2 | 180 |
| User Feedback/Testing | 4 | 10 | 1 | User testing/talk through sessions using website prototype, 10 individual 1-1 sessions with users between Mar 11-21 | 60 | 10 | 600 |
| Web Development 1 | 5 | 0 | 1 | Mission Vision | 60 | 1 | 60 |
| Web Development 2 | 4 | 0 | 1 | Brand Strategy | 60 | 1 | 60 |
| Web Development 3 | 3 | 0 | 1 | Look and Feel | 60 | 1 | 60 |
| Web Development 4 | 3 | 0 | 1 | Details | 60 | 1 | 60 |
| Web Development Meeting 5 | 1 | 0 | 1 | Details 2 | 60 | 1 | 60 |
| Web Development 6 | 3 | 0 | 1 | Details 3 and Content | 60 | 1 | 60 |
| Content Development Meetings | 5 | 0 | 0 | 5 meetings to coordinate content development for website pages, with internal team of 5 | 45 | 5 | 225 |

Supplementary Table 2: Summary of meeting frequency and duration during the co-design phase

## 2.4 Supplementary Table 3: Raw qualitative data from open-ended evaluation survey questions

**What did you like about Just One Move? Why?
31 responses, 6 blanks**

- Usability
  - Visual Appeal
    - *I like the flow of information, the use of movement, and the colours.*
    - *The use of clear headings and spacing made it easy to find the information I was looking for without feeling overwhelmed.*
    - *Nice graphic and large enough fonts.*
    - *Also like that the models are real people that see to suffer from arthritis. Age appropriate and relatable.*
    - *Clear and easy to read*
    - *Small blocks of text instead of walls of text.*
    - *Ease of use and attractive color*
    - *I like the layout and graphics. It’s well designed and attractive.*
  - Ease of Navigation/Use
    - *As a retired software product manager, I would say this is a solid starting point and can be tweaked and improved over time with feedback. Overall very good job.*
    - *I like it easy to navigate, easy to follow.*
    - *Main Pages shows the Overall of each section by scrolling down the page you can click on the section that is appropriate for you that day.*
    - *It’s attractive and easy to navigate*
    - *I liked how clean and easy to navigate the website is. The layout is intuitive, and pages load quickly.*
    - *Ease of use and clarity/visual effects of the content and navigation*
    - *Seems easy to navigate and visually easy to read.*
    - *Easy to look at, well laid out, many resources, community aspect.*
    - *Easy to flow through easy to understand great*
    - *Easy to read and follow, the navigation is good and the information is setup well.*
    - *Navigation was easy, the drop downs had clear options to move throughout the site. Relevant contact and site information easy to find at the bottom.*
    - *Very clean UX!*
- Valuable Content/Usefulness
  - Inspiring confidence to move for people with RA
    - *It would be useful for someone that hasn’t been active at all with RA*
    - *It gives confidence for anyone to take their physical exercise serious no matter the challenges stopping them from exercising*
    - *The website could be very helpful for people with RA if you could get the word out!*
    - *I think it was basically satisfactory. Encouraged me to exercise, cited specific benefits, offered a variety of comments and encouragement*
  - A space for community support
    - *I loved that other people like myself that have arthritis have a platform to share their experience, passion, knowledge of arthritis. I love that.*
    - *I really love how this website allows me to share my little wins and see other people's stories. It’s so inspiring! The tips and motivation available here keep me going and push me to get better physically every day.*
    - *I like the profiles of people living with RA and how they incorporate physical activities*
  - Practical tips to get moving
    - *That there is one with good general information*
    - *It seemed relevant and positive, gave good reasons for being physically active even if my body has some pain.*
    - *It's engaging and has lots of information. I like that you can customize it for yourself.*
    - *Lots of info*
    - *Many ideas and info with help and examples.*
  - Making movement approachable through small steps
    - *Simple moves with repetition. Gives achievable goals this way*
    - *Variety of different activities to try in order to maintain movement.*
    - *The variety of exercises. Provided things that I had not thought of or would be to hard on my joints. They have been modified to help those with RA*
    - *The choice of length of exercises / movement - Single Move, 3-5 moves or Many Moves. Can therefore vary it each day.*
    - *I liked the personal examples of different types of exercise and their specific effects*
    - *Easy to input goals, and print it out for accountability. Ability to develop a program, incorporating key elements - Strength, balance/coordination, range of motion/flexibility*
  - Clear written and video instructions to practice movements
    - *I like the exercise videos*
    - *Option for video which is so motivational. Hate just video as when travelling, that is an issue.*
    - *Simple, easy to follow instructions with video showing how to do the exercises*
- Negative Experience
  - *What did you like? Nothing*

**What did you not like about Just One Move?
31 responses, 6 blanks**

**What could we improve?
24 responses, 13 blanks**

- Nothing to dislike
  - *Nothing dislike*
  - *I do not see anything that challenged me to search, I like this Website*
  - *Nothing*
  - *There's nothing I don't Ike about the website*
  - *Nothing so far*
  - *None*
  - *I believe I need to use it more but at first work through, there is nothing I don't like.*
  - *nothing to dislike*
  - *There was nothing that I didn't like!*
- No improvements needed
  - *All good, nothing to change.*
  - *No concerns that I could see for Improvement*
  - *n/a*
  - *Everything is appropriate and complete.*
  - *None*
- Ease of use challenges
  - *Not clear how to use it*
  - *Without my daughter's help I couldn't use the site.*
  - *Navigating the site. It jumps from number 1 to non-sequential numbers. It makes you add in your name. Have to back out just to look at it. Needs a Topic button/ bar to see what you want to go to next*
  - *Menu buttons could be a little bigger. Especially for someone with poor dexterity*
  - *I didn't realise until 15 minutes into exploring that I could have "favourites" without having an account - so I was exploring how to make an account.*
  - *It took me quite a while of looking around the site to understand how it works. It was not intuitive for me.*
  - Mobile Interface
    - *This made those pages less appealing and harder to scan quickly. Additionally, on mobile, a few buttons were too small or close together.*
    - *I tried to access it on my phone and it was not as easily viewable as using it desktop.*
- Ease of use improvements
  - *A home button at the top of the page would make navigation easier for older users.*
  - Mobile experience
    - *I’d love to see improvements in mobile responsiveness—especially button spacing and font sizing.*
    - *Mobile app: A handy app versus printing off where I’m motivated to use it because it shows the progress both good and bad and encouraging to keep me wanting to enter data*
  - Revamp filter functionality in the movement vault
    - *The filter is visually confusing, a regular drop-down or check boxes would make this more navigable.*
- Onboarding and guidance to orient users
  - *Adding short explainer videos or icons in information-heavy sections would also enhance engagement and understanding.*
  - *Directions*
  - *I think the home page needs to be About Us as that page gives a good overview of what you are trying to achieve and helping those affected with RA.*
  - *Also Habit Builder does not have a drop down menu - perhaps a few words to describe how you help i.e. weekly motivational email.*
  - *In the Habit Builder section - clearer explanation on what is happening might be helpful - talk about building the plan.*
  - *I would make the Just One Move logo on the main landing page larger. It blends in with the menu options to the right and should stand out as the name of the site.*
- Presentation of Information: Too much information can feel overwhelming
  - *Some sections felt a bit text-heavy or lacked engaging visuals.*
  - *There is alot of information that could make me feel overwhelmed. Lots of things to read.*
  - *It is fast paced in changing from this, to that, and that! Changes in front of my eyes from one page to another before I can read what I have on my screen! That put me off! At first.*
  - *That I can’t just speak my response*
- Presentation of information: accessibility and readability improvements
  - *Stop changing so fast from one page to another!*
  - *In general, a lot of the text is totally flush with the left side of the monitor. A bit of blank space between the edge of the monitor and the text would make this easier to read.*
  - *Nice if I could just voice activate that would be something great that some tracking apps have*
  - *A dark mode option could be a nice touch for users browsing at night.*
  - *Specific wording changes*
    - *I didn’t like the word Snacks used. Thought it was an eating part of App. I’m on a diet program and don’t want to be reminded of food while trying to exercise.*
- Lack of interest in content or limited value
  - *I don’t really like to read about other people’s experiences.*
  - *So many personal stories. I’m not as interested in that as I always question the validity of personal stories.*
  - *Because I am already physically active and reasonably fit, and access a different exercise regime, it didn't really increase my knowledge or motivation.*
- Limited ability to interact or personalize the experience
  - *Did not see where community could interact. One of the survey questions asked about configurability, but I did not notice any ability to configure.*
- Repetitive content and related errors
  - *There were places where the same exercise came up again and one exercise that I couldn’t get to open up*
- Weekly content from health professionals
  - *I think having certified coaches or health professionals share weekly tips or Q&As could add more value and trust.*
- Expanded collection of movements to choose from
  - *Movement vault is limited, especially when you select 15-30 mins. nothing appears*
  - *A few of the exercises have exactly the same movements and repetitions under easy, medium and hard. For example - shoulder Rolls and arm circles, under just one movement.*
  - *Have a video with all of the exercises*
  - *MORE exercises!*
- AI images reduce sense of connection and credibility
  - *I did not like the use of AI images. The colours used in the images work nicely overall with the cohesiveness of the site. But the AI people are creepy to me and don't make me feel like the site real or for real people.*
  - *Some of the AI images are off, people missing limbs or looking strange. Maybe use images of real people.*
  - *Didn't love the big pictures of people in green shirts*
- Replace AI images with real people
  - *Again, use of AI images are not perfect.*

Supplementary Table 3: Raw qualitative data from open-ended evaluation survey questions

# 3 Supplementary Figures

## 3.1 Supplementary Figure 1: Screenshot of the initial intervention prototype in Microsoft PowerPoint

Supplementary Figure 1: Screenshot of the initial intervention prototype in Microsoft PowerPoint.
